# An Explanation for the Adiponectin Paradox

**DOI:** 10.3390/ph14121266

**Published:** 2021-12-04

**Authors:** Hans O. Kalkman

**Affiliations:** Gänsbühlgartenweg 7, 4132 Muttenz, Switzerland; hans.kalkman@bluewin.ch; Tel.: +416-1362-0110

**Keywords:** insulin resistance, glycosyl phosphatidylinositol-phospholipase D, type 2 diabetes, T-cadherin, hyperadiponectinemia

## Abstract

The adipokine adiponectin improves insulin sensitivity. Functional signal transduction of adiponectin requires at least one of the receptors AdipoR1 or AdipoR2, but additionally the glycosyl phosphatidylinositol-anchored molecule, T-cadherin. Overnutrition causes a reduction in adiponectin synthesis and an increase in the circulating levels of the enzyme glycosyl phosphatidylinositol-phospholipase D (GPI-PLD). GPI-PLD promotes the hydrolysis of T-cadherin. The functional consequence of T-cadherin hydrolysis is a reduction in adiponectin sequestration by responsive tissues, an augmentation of adiponectin levels in circulation and a (further) reduction in signal transduction. This process creates the paradoxical situation that adiponectin levels are augmented, whereas the adiponectin signal transduction and insulin sensitivity remain strongly impaired. Although both hypoadiponectinemia and hyperadiponectinemia reflect a situation of insulin resistance, the treatments are likely to be different.

## 1. Introduction

Healthy adipose tissue produces a protein called adiponectin that displays anti-inflammatory activity in macrophages [1,2,3] and improves insulin sensitivity in skeletal muscle [4]. Overnutrition with calory-rich foods leads a hypertrophy of abdominal and subcutaneous white adipose tissue [5]. Hypertrophic white adipose tissue is poorly perfused and the resulting hypoxia shuts down the production of adiponectin [6,7]. Overnutrition-induced adipocyte hypertrophy thereby promotes the development of insulin resistance, chronic inflammation, macrophage infiltration into the adipose tissue, mitochondrial dysfunction and ultimately adipocyte death [8,9]. These negative consequences of overnutrition are reversed by drastic reduction in food intake [6,10], as well as by bariatric surgery [8,11,12,13,14], and are accompanied by a normalization of serum adiponectin levels [7,10,11,12,13]. Consequently, hypoadiponectinemia is considered both a risk factor [15,16] and a biomarker [17,18,19] of type 2 diabetes. In a meta-analysis of 34 prospective studies [20], low adiponectin levels were associated with an increased risk of developing type 2 diabetes. Whilst obesity usually gives rise to an increase in blood pro-inflammatory cytokines [21,22] and an altered lipid profile [23,24], the association between low levels of adiponectin and type 2 diabetes remained unchanged after adjusting for inflammation and dyslipidemia markers [25]. The association was, however, substantially attenuated after adjustment for insulin sensitivity or glycemia markers. This result suggests that it is the insulin sensitivity not the obesity that drives the association between adiponectin and type 2 diabetes [20].

## 2. Adiponectin Levels in Metabolic Syndrome

Based on these clinical studies and the mechanistic studies in animals, one would anticipate that hypoadiponectinemia would be typical for patients with insulin resistance (including metabolic syndrome, as well as related diseases such as atherosclerosis, cancer, vascular depression and dementia) [25,26,27]; however, the reality is different. Rather than hypoadiponectinemia, numerous studies report elevated adiponectin levels. For instance, type 2 diabetic patients with genetically defective insulin receptors or insulin resistance due to antibodies against the insulin receptor display remarkably high adiponectin levels [28]. In patients with type 1 diabetes, the circulating levels of adiponectin are also increased [29]. In the latter study, it is remarkable that adiponectin was mainly of the high molecular weight form (see below), whereas there was evidence for insulin resistance [29]. Moreover, cardiovascular mortality in patients with type 2 diabetes did not correlate with hypoadiponectinemia as expected but counterintuitively with high adiponectin levels [30]. Defective insulin signaling is also a hallmark of cognitive decline, mild cognitive impairment and Alzheimer’s disease [31,32,33]. Nevertheless, studies that investigated plasma and CSF levels of patients with dementia invariably observed increases rather than the expected decreases in adiponectin [34,35,36,37,38]. Finally, although a positive association exists between insulin resistance and depression [39], there are reports that high levels of adiponectin are associated with higher depression scores [40,41].

The situation where high adiponectin levels do not translate into beneficial insulin sensitivity and health benefits is often referred to as “the adiponectin paradox” [28,30,42,43,44]. Over the years, several biological mechanisms have been proposed. These included renal dysfunction, decreased hepatic clearance, a compensatory rise in adiponectin in response to subclinical pathology or a response to sarcopenia [45]. None of these proposed mechanisms has been definitely proven or refuted. In a study by Uetani et al. [46], the authors controlled for renal function and sarcopenia, which indicates that these two factors are unlikely to explain the “adiponectin paradox”. Based on recent insights, an alternative potential explanation for the adiponectin paradox is proposed in the current manuscript.

## 3. Adiponectin Signaling

In blood, adiponectin circulates as trimers, hexamers and as a ‘high molecular weight (HMW)” polymer of 12–18 units [25]. Three cell-membrane proteins (T-cadherin, AdipoR1 and AdipoR2) bind adiponectin, but only the latter two provide intracellular signal transduction [3,15,47,48]. The hexamer and HMW forms of adiponectin display the highest affinity for T-cadherin [49], whereas the trimeric and smaller forms of adiponectin preferentially bind AdipoR1 and AdipoR2 [25]. T-cadherin is a glycosyl phosphatidyl-inositol (“GPI”)-anchored cell membrane protein that lacks an intracellular domain [50]. The observation that the distribution of T-cadherin largely overlaps with that of the AdipoRs [16] supports the notion that T-cadherin acts as a co-receptor for the AdipoRs [25,50]. In the absence of T-cadherin (for instance after genetic knockdown or knockout), adiponectin production remained unaffected, although the protein was no longer accumulated by the tissues and the levels of adiponectin (hexamers, HMW multimers) in the circulation were augmented [51]. Apparently, T-cadherin is essential for signal transduction because it sequesters hexamer and HMW-adiponectin from the circulation to enable physiologically relevant concentrations in the proximity of the two AdipoRs [51]. High molecular weight adiponectin, together with T-cadherin, is endocytosed into multivesicular bodies of T-cadherin-expressing cells and subsequently released into the extracellular space as exosomal cargo together with T-cadherin and ceramide [52]. Adiponectin enhanced exosome production and secretion and simultaneously reduced the intracellular levels of ceramide [52]. This process, which is adiponectin- and T-cadherin-dependent but AdipoR-independent, probably plays a role in organ protection [52]. Ultimately, circulating exosomes and their cargo are phagocytosed and metabolized by hepatic macrophages [52]. An important point is that the removal of intracellular ceramides improves insulin sensitivity [53,54,55]. In addition to exosome production, adiponectin also improves insulin function via a signaling pathway that involves the AdipoR1 receptor. This pathway ultimately results in stimulation of AMP-activated protein kinase (AMPK), which in turn increases glucose uptake [56].

## 4. Mechanistic Explanation for the “Adiponectin Paradox”

T-cadherin deficiency in animal studies strongly diminished the functional response to adiponectin, while concomitantly producing a “dramatic” increase in circulating levels of HMW-adiponectin [50,51]. This is reminiscent of clinical studies that reported high levels of HMW-adiponectin in the absence of evident beneficial health effects (sometimes referred to as “adiponectin resistance” [29,30]). As mentioned above, T-cadherin is anchored to the cell membrane via its glycosyl phosphatidylinositol moiety and can be cleaved by a specific phospholipase D (GPI-PLD) [57]. During diet-induced obesity or insulin resistance, the circulating levels of GPI-PLD are significantly augmented, which potentially increases the cleavage of T-cadherin [58]. The mechanism via which obesity enhances the hepatic expression and circulating levels of GPI-PLD is not known, but it seems independent of plasma insulin levels [58]. Under such circumstances the adiponectin-sequestering action of T-cadherin would be lost and AdipoR signaling would be impaired [51]. There is indirect support for this scenario in the clinical literature. Polymorphisms in the gene that encodes T-cadherin (CDH13) are known to influence the baseline values of HMW-adiponectin [46,59]. It is conceivable that these polymorphisms affect the function and stability of T-cadherin. Furthermore, GPI-PLD-mediated cleavage of T-cadherin should lead to measurable levels of “soluble” T-cadherin. Soluble T-cadherin is indeed found in human blood, where it occurs in a 130-KDa, a 100-KDa and a 30-KDa form. The levels of the 30-kDa segment in type 2 diabetic patients were associated with clinical parameters, such as the duration of diabetes, HbA1c, serum C-peptide and LDL-cholesterol [60]. Importantly, the levels of GPI-PLD seem to be influenced by adiponectin, since functional adiponectin signaling suppresses plasma GPI-PLD levels [51]. Dysfunctional adiponectin signaling, as caused by overnutrition, might trigger a vicious process where insufficient adiponectin signal transduction raises the amount of GPI-PLD, causing a loss of membrane-bound T-cadherin, and consequently a further decline in adiponectin signal transduction. Indeed, in humans, plasma GPI-PLD levels correlate with insulin resistance, triglyceride levels and non-alcoholic fatty liver disease [58,61,62]. When HMW-adiponectin is no longer sequestered by the tissues, the levels in blood will rise. Therefore, the enzymatic removal of T-cadherin from the cell surface also provides an explanation for the paradoxical rise in HMW-adiponectin levels in the circulation.

## 5. Discussion

It should be noted that the current theory for the “adiponectin paradox” remains a theory and should be scrutinized by experiments. There are two potential weak points to the theory. In the first place, the notion that adiponectin positively influences the levels of T-cadherin is presently supported by a single publication only [51], so independent confirmation would be useful. Two groups [58,63] have reported that diabetes causes an increase in GPI-PLD activity and a decrease in tissue T-cadherin levels [51]. Since diabetes is initially associated with a *decreased* production of adiponectin by adipocytes [15,16], this could explain the *increase* in GPI-PLD noted by Musada et al. [58] and Müller et al. [63], and this in turn would explain the *loss* of membrane-bound T-cadherin reported by Matsuda et al. [51]. Thus, the existing literature is internally consistent. The second point concerns the co-localization of AdipoR and T-cadherin [16,25,51]. A complete co-localization is contested by Kadowaki et al. [15], who noted that hepatocytes, an important target for insulin-mediated glucose metabolism, do not express T-cadherin. In this respect, it should be noted that GPI-PLD is abundantly expressed in hepatocytes [58,64]. In these cells, GPI-PLD causes the production of diacylglycerol (from glycosyl phosphatidylinositol), which activates protein kinase Cε. This kinase phosphorylates insulin receptor substrate-1, which inhibits insulin signaling [58]. Therefore, in hepatocytes it is the activation of GPI-PLD that causes the insulin resistance, not the hypoadiponectinemia. Concerning the hepatocytes, it seems that adiponectin and T-cadherin play only minor roles. Hyperadiponectinemia with HMW-adiponectin is also seen in chronic heart failure [30,65] and in autoimmune diseases [66,67]. In autoimmune diseases, high circulating levels of TNFα [68] suppress the adiponectin secretion by adipocytes [10,69,70] and predict the development of type 2 diabetes [71,72]. The consequence would be an increase in GPI-PLD (as described by Masuda et al. [58]), followed by hydrolysis of T-cadherin from the cell membrane and an increase in HMW-adiponectin circulation. In heart failure, hypertrophic cardiomyocytes release atrial natriuretic peptide, which activates adiponectin production by adipocytes [73]. Cellular hypertrophy (here cardiomyocytes) is associated with an increased release of GPI-anchored proteins [63], presumably including T-cadherin. This could be the trigger for an increase in GPI-PLD activity, further loss of membrane-bound T-cadherin and a poor sequestration of HMW-adiponectin from the circulation. Consistent with this notion, treatment with diuretics and vasodilators in order to limit cardiomyocyte hypertrophy provoked a reduction in hyperadiponectinemia in heart failure patients [74].

If the above outlined vicious process is validated by additional experiments, it would provide strong support for the notion that both low and high plasma levels of adiponectin reflect a state of insulin resistance. Hypoadiponectinemia would be more benign and could be corrected by treatments that raise the production of adiponectin (for instance fish oil [75,76], abnormal cannabidiol [77], thiazolidinediones [78] or natriuretic peptides [73]). Such therapeutic approaches would work only when the obligatory co-receptor T-cadherin is still present. In contrast, insulin resistance plus hyperadiponectinemia (and absence of membrane-bound T-cadherin) would require AdipoR agonists such as AdipoRon [79,80,81] that act independently of the co-receptor T-cadherin, or alternatively compounds such metformin (see below) that directly activate the intracellular signaling pathway.

**Figure 1:**
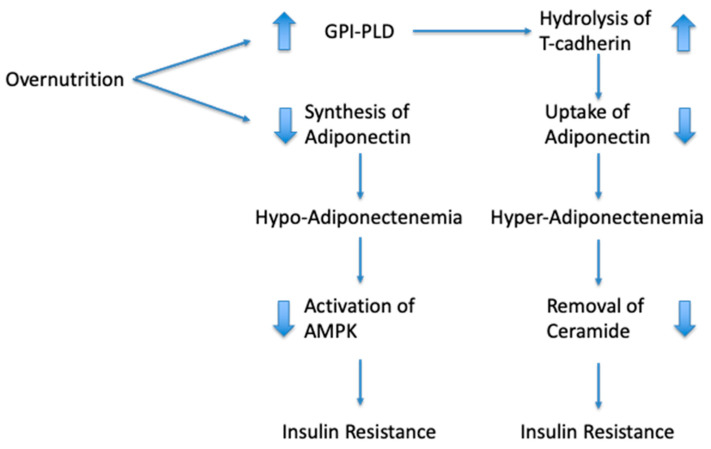
Schematic representation of the association between overnutrition and circulating levels of adiponectin. Overnutrition provokes adipocyte hypertrophy, which gives rise to hypoxia and a reduction in adiponectin synthesis. Dysfunctional adiponectin signaling causes a reduction in AMPK activity, which contributes to insulin resistance. Overnutrition also increases the production of GPI-PLD and its levels in circulation. The GPI-PLD enzyme mediates hydrolysis of T-cadherin. When adiponectin is absent from the cell surface, the cells are no longer able to sequester adiponectin and adiponectin levels in blood become elevated. Cells sequester adiponectin and T-cadherin to allow the disposal of ceramides. Dysfunctional ceramide disposal is a further cause of insulin resistance.

The signaling pathway downstream of the receptors AdipoR1 and AdipoR2 involves an adaptor protein (adaptor protein, phosphotyrosine interacting with PH domain and leucine zipper 1; APPL1), as well as the serine/threonine kinase, liver kinase B1 (LKB1) [25,82]. LBK1 phosphorylates and activates AMP-activated protein kinase (AMPK) [82,83]. AMPK is an enzyme that responds to a low ATP/AMP ratio (signifying a low energy state). Activation of AMPK stimulates processes that aim to increase ATP [83]. These include increased glucose transport, mitochondrial biosynthesis and fatty acid oxidation, while inhibiting the synthesis of fatty acids, proteins and glycogen [83]. When the adiponectin signal transduction pathway is functional, it leads to AMPK activation and provokes a shift away from energy storage towards ATP production and energy dissipation (reducing white adipose fat tissue and obesity). Adiponectin indirectly restores insulin sensitivity by inducing fatty acid oxidation and reducing free fatty acid levels and ectopic lipid deposition [25,38]. Therefore, adiponectin-induced activation of AMPK helps to limit the consequences of overnutrition, thereby indirectly helping to restore insulin sensitivity (see Figure 1). Metformin is currently the only registered compound that acts on the adiponectin pathway underneath the adiponectin receptors. It increases the kinase activity of LKB1 and induces AMPK activation [82,84,85]. At present, metformin is the therapy of choice in patients suffering from insulin resistance with “paradoxical” hyperadiponectinemia.

In summary, overnutrition causes a reduction in adiponectin synthesis and is associated with an increase in the circulating levels of GPI-PLD. GPI-PLD promotes the hydrolysis of membrane-anchored T-cadherin, which has a detrimental effect on the sequestration of HMW-adiponectin from the blood. HMW-adiponectin in conjunction with T-cadherin is responsible for the removal of cellular ceramides, and dysfunction of this process causes a further worsening of the insulin resistance. Consequently, both low and high levels of adiponectin reflect a state of insulin resistance but require different treatments.

## Data Availability

Not applicable.
